# Feasibility of sentinel lymph node mapping in ovarian tumors: A systematic review and meta-analysis of the literature

**DOI:** 10.3389/fmed.2022.950717

**Published:** 2022-08-01

**Authors:** Saeideh Ataei Nakhaei, Sayyed Mostafa Mostafavi, Marjaneh Farazestanian, Malihe Hassanzadeh, Ramin Sadeghi

**Affiliations:** ^1^Nuclear Medicine Research Center, Mashhad University of Medical Sciences, Mashhad, Iran; ^2^Department of Artificial Intelligence, School of Computer Engineering, University of Isfahan, Isfahan, Iran; ^3^Women’s Health Research Center, Mashhad University of Medical Sciences, Mashhad, Iran

**Keywords:** sentinel node, lymphatic mapping, meta-analysis, systematic review, ovarian cancer, nuclear medicine, lymphoscintigraphy

## Abstract

**Purpose of the report:**

Since the presence of lymph node metastases upstages the disease and to reduce the morbidity of total lymphadenectomy, sentinel lymph node (SLN) mapping in ovarian mass has been the focus of extensive research. This study aims to review all the literature associated with ovarian SLN mapping and assess the feasibility of ovarian SLN mapping.

**Materials and methods:**

PubMed and Scopus were searched using the following keywords: (Sentinel lymph node) AND (Ovary OR Ovarian) AND (Tumor OR Neoplasm OR Cancer). All studies with information regarding sentinel node biopsy in ovaries were included. Different information including mapping material, injection sites, etc., was extracted from each study. In total, two indices were calculated for included studies: detection rate and false-negative rate. Meta-analysis was conducted using Meta-MUMS software. Pooled detection rate, sensitivity, heterogeneity, and publication bias were evaluated. Quality of the studies was evaluated using the Oxford center for evidence-based medicine checklist.

**Results:**

Overall, the systematic review included 14 studies. Ovarian SLN detection rate can vary depending on the type of tracer, site of injection, etc., which signifies an overall pooled detection rate of 86% [95% CI: 75–93]. The forest plot of detection rate pooling is provided (Cochrane Q-value = 31.57, *p* = 0.003; I^2^ = 58.8%). Trim and fill method resulted in trimming of 7 studies, which decreased the pooled detection rate to 79.1% [95% CI: 67.1–87.5]. Overall, pooled sensitivity was 91% [59–100] (Cochrane Q-value = 3.93; *p* = 0.41; I^2^ = 0%). The proportion of lymph node positive patients was 0–25% in these studies with overall 14.28%.

**Conclusion:**

Sentinel lymph node mapping in ovarian tumors is feasible and seems to have high sensitivity for detection of lymph node involvement in ovarian malignant tumors. Mapping material, injection site, and previous ovarian surgery were associated with successful mapping. Larger studies are needed to better evaluate the sensitivity of this procedure in ovarian malignancies.

## Introduction

Ovarian cancer is one of the leading causes of gynecological cancer-related mortalities. This is primarily due to the fact that 75% of cases are diagnosed in advanced stages of the disease. However, up to one-third of patients with ovarian cancer are diagnosed in early stages of the disease (1). Because of missing involved lymph nodes during lymph node sampling, bilateral pelvic and para-aortic lymphadenectomy is the N-staging recommendation by FIGO in early stage ovarian cancer (2). However, only 14% (3) of these patients have nodal metastasis and would not benefit should routine lymph node dissection be performed. Additionally, systematic lymphadenectomy has a high risk of complications such as longer operative time, longer hospital stay, larger amount of blood loss, and an increased risk of developing lymphoceles or chronic lower limb lymphedema (4–6). Because the occurrence of lymph node metastases increases the severity of the disease (7) and in an attempt to diminish procedure-related morbidity of routine systematic lymphadenectomy, sentinel lymph node (SLN) mapping in ovarian mass has been the focus of extensive research over the past decade. The aim of this study was to systematically review the available series on sentinel lymph node mapping in ovarian mass. This includes a discussion of the feasibility of ovarian SLN mapping and a characterization of these studies.

## Materials and methods

We searched PubMed and Scopus for relevant articles with following keywords on 6 October 2021:

(Sentinel lymph node) AND (Ovary OR Ovarian) AND (Tumor OR Neoplasm OR Cancer).

A number of two reviewers (SA and RS) jointly examined the retrieved articles by going through the title and abstract sections. All studies with information regarding sentinel node biopsy in ovaries (ovarian tumors or normal ovaries) were included into the systematic review. The reference lists as well as citing articles of included studies were searched for possible missing relevant studies. Citing articles were evaluated using Google Scholar. Case reports (less than 5 patients), review articles, and commentaries were excluded. No language restriction was imposed on our search.

Information on mapping material, injection sites, time from injection to sentinel node mapping, number and location of sentinel nodes, lymphoscintigraphy findings, pathological involvement of the sentinel nodes, and other harvested nodes was extracted from each included study by the same two reviewers. This was done by going through the Methods and Results sections of the studies. A number of two indices were calculated for included studies: Detection rate: number of patients with at least one detected sentinel node/all patients. We also performed a sensitivity analysis for studies which only included ovarian tumors.

False-negative rate: number of patients with pathologically involved non-sentinel node despite non-involved sentinel node/all patients with at least one detected sentinel node.

Meta-analysis was done using Meta-MUMS software (8). Random effects model was used for pooling data across included studies. Forest plots were used for visual presentation of the results. Heterogeneity was evaluated using Cochrane Q-value (*p* < 0.05 was considered statistically significant) and I2index. Subgroup analysis was used to explore the heterogeneity across studies. Funnel plots and Egger’s regression intercept were used to evaluate possible publication bias.

Quality of the included studies was evaluated by Oxford center for evidence-based medicine checklist for diagnostic studies^1^. This review was prepared according to PRISMA checklist^2^.

## Results

**FIGURE 1:**
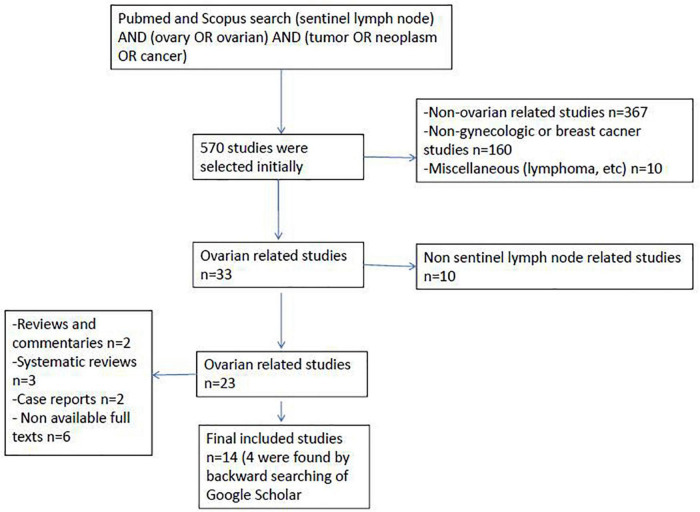
Flow chart of the study selection.

**FIGURE 2:**
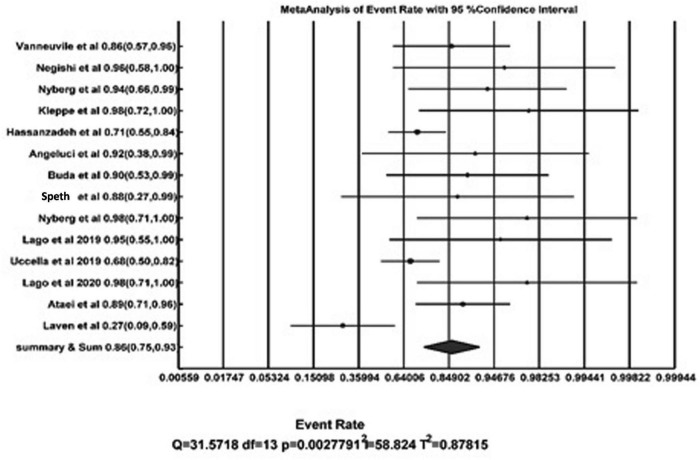
Forest plot of detection rate pooling.

**FIGURE 3:**
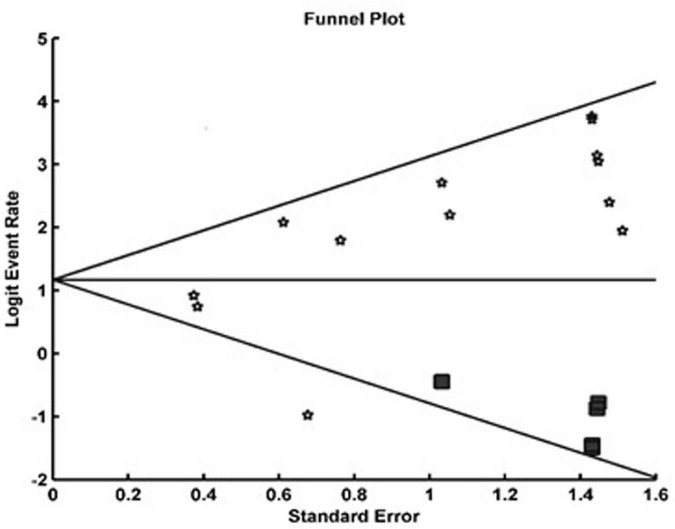
Funnel plot of detection rate pooling using trim and fill method.

We identified a total of 14 sentinel lymph node ovarian-related studies (Figure 1). Overall, a total of 234 patients were included in the studies who underwent sentinel lymph node ovarian mapping (Table 1). A total of 162 (69%) patients underwent laparotomy whereas the remaining 31% of patients underwent minimal invasive surgery (laparoscopy). The majority of the patients (81%) had suspicious ovarian mass (121 patients) or early ovarian cancer (69 patients). Other cases of note were other gynecological cancer (29 endometrial cancer, 3 cervical cancer, and 1 fallopian tube tumor) identified for surgery. Ovarian sentinel lymph nodes detection rate can vary depending on the type of tracer, site of injection, etc. Sentinel lymph nodes were found in 199 of 234 patients, which signifies an overall pooled detection rate of 86% [95% CI: 75–93]. Figure 2 shows the forest plot of detection rate pooling (Cochrane Q-value = 31.57, *p* = 0.003; I^2^ = 58.8%). Figure 3 shows the funnel plot of the overall detection rate pooling. Trim and fill method after trimming 7 studies decreased the pooled detection rate to 79.1% [95% CI: 67.1–87.5]. Table 2 shows the subgroup analyses of the detection rate pooling according to different variables.

**TABLE 1:** Characteristics of the studies (*N* = 14).

First Author/Year	Country under study	Study Population	Number of patients	LPS vs. LPT	Mapping Material	Tracer dosage	Site of injection	Wait time after injection	SLN identification criteria	Detection Rate/False-negative Rate	Quality assessment according to OCEBM			

											Consecutive recruitment	Gold standard	Enough explanation of the test	Application of gold standard to all patient regardless of SLN results
Vanneuville et al. (22)	France	Ablation of benign ovarian cyst or for tubal ligation	14	LPS	Tc-99m + rhenium sulfide colloid	37 MBq (1 mci) + 0.5– 0.7 mL	Mesovarium (of normal ovaries)	4-6 h (scintigraphy, not intraoperative gamma probing)		85.7%/NA	NA	NA	Yes	NA
Negishi et al. (13)	Japan	Ten endometrial cancer, one Fallopian tube tumor	11	LPT	CH40 (charcoal solution)	0.05–0.2 mL	Ovarian cortex	10min	“visual identification”	100%/NA	Yes	LND Only for malignant cases	Yes	Yes
Nyberg et al. (14)	Finland	High-risk endometrial carcinoma	16	LPT	Tc-99m albumin nanocolloid + Blue dye	0.8 mL + 2 mL	Hilum of the ovary (8 right, 8 left)	Minimum 10 min	“hot” node/10 fold	94%/NA	Yes	LND	Yes	Yes
Kleppe et al. (15)	The Netherlands	patients with a pelvic mass suggestive of a malignant ovarian tumor	21	LPT	Tc-99m albumin nanocolloid + Blue dye	0.5 mL + 2 mL	Proper ovarian and suspensory ligament	Minimum 15 min	At least 10 fold	100%/0%	NA	LND Only for malignant cases	Yes	Yes
Hassanzadh et al. (9)	Iran	patients with ovarian mass (cancer = 13,benign = 1, borderline = 21 patients)	35	LPT	Tc-99mPhytate + Blue dye (in only four patients)	0.4 mL + 0.4 mL	10: normal ovarian cortex 25: proper ovarian and suspensory ligament	10 min	“true SLN”/at least 3 fold	Cortex injection: 40%/0% ligaments injection: 84%/0% Radiotracer 71.4%/0%	Yes	LND Only for malignant cases	Yes	Yes
Buda et al. (21)	Italy	Suspicion of malignant ovarian tumor (7 patients) + cervical carcinoma (3 patients)	10	LPS	ICG	0.5–1 mL (125 mg/mL)	Dorsal and ventral side of the proper ovarian and suspensory ligament	Real time		90%/NA	Yes	LND Only for malignant cases	Yes	All but one case
Speth (16)	Italy	Three endometrial cancer G3	3*	LPT	Tc-99m albumin nanocolloid + Blue dye	80 MBq (2 mci) + 0.2– 0.5 mL	Proper ovarian and suspensory ligament	15 min	At least 10 fold	100%/NA	NA	LND	Yes	Yes
Nyberg et al. (17)	Finland	ovarian mass (cancer = 5,benign = 11, borderline = 4 patients)	20	LPT	Tc-99m albumin nanocolloid + Blue dye	1 mL + 2 mL	Under the serosa, next to the junction of the ovarian tumor (mesovarium)	10-20 min		100%/0%	NA	LND Only for malignant cases	Yes	Yes
Lago et al. (10)	Spain	Early ovarian cancer	10	LPS (3 patients)and LPT (7 patients)	Tc-99m albumin colloid + IGC	37 Mbq (1 mci) + 0.5 mL	Proper ovarian and suspensory ligament stumps	15-30 min	10 fold	Tc-99m radiocolloid: 100% (IGC: 90%)/50%	NA	LND	Yes	Yes
Uccella et al. (18)	Italy	Early ovarian cancer	31	LPS	ICG	2 mL	Dorsal and ventral side of the proper ovarian and suspensory ligament	5-20 min		67.7%/0% (First surgery:88.9% Re-staging:38.5%)	Yes	LND	Yes	Yes
Lago et al. (11)	Spain	Early ovarian cancer	20	LPS (9 patients) and LPT (11 patients)	Tc-99m albumin colloid + IGC	37 Mbq(1mci) + 0.5 mL	Proper ovarian and suspensory ligament stumps	15-30 min	“hottest SLN”	Tc-99m radiocolloid:100%/ NA IGC: 95%/NA	Yes	LND	Yes	Yes
Laven et al. (12)	Netherlands	pelvic mass suspicious for malignancy (8 patient) or with history of prior resection of a malignant ovarian mass (3 patient)	11	LPT	Tc-99m albumin nanocolloid + Blue dye	20 Mbq (0.5 mci) + 0.2 mL	dorsal and ventral sides of the remains of the proper ovarian and suspensory ligaments	At least 15 min		Tc-99m:27%/NA Blue dye: 0%/NA	NA	LND Only for malignant cases	Yes	Yes
Ataei et al. (19)	Iran	Suspicion of malignant ovarian tumor	27	LPT	Tc-99m fytate	18.5 MBq (0.5 mci)	proper ovarian and suspensory ligament	15-20 min		89%/NA	Yes	LND Only for malignant cases	NA	Yes

*8 patients were considered in this study. Of these, 5 cases with an ovarian tumor were published elsewhere (15). As such, these 5 patients were excluded from the study in question. ICG, indocyanine green; LPS, laparoscopy; LPT, laparotomy; Tc-99m, technetium 99; NA, not available; SPECT/CT, single-photon emission computed tomography/computed tomography; OCEBM, Oxford center for evidence based medicine; LND, lymph node dissection.

**TABLE 2:** Pooled detection rate based on different variables.

Variable	Pooled detection rate [95% confidence interval]	
Overall	86% [75–93]	
Mapping material	Tc-99m radiocolloid	81% [66–91]
	Tc-99m radiocolloid and Blue dye	95% [84–99]
	Indocyanine green	84% [67–93]
	Blue dye	60%[44–73]
Injection site	Cortex	62% [26–88]
	Mesovarium	91% [67–98]
	Hilum	93%[67–99]
	Ligament	85% [71–92]
	Stump	59% [32–81]
Ovary with underlying pathology or normal	Ovary with mass	82% [70–89]
	Normal ovary	91% [75–97]
Procedure (LPT vs. LPS)	Laparoscopy (LPS)	80% [61–91]
	Laparotomy (LPT)	88% [77–95]

Analysis after the exclusion of cases with endometrial carcinoma, cervical carcinoma, and fallopian tube tumors showed pooled DR of 78[95% CI: 60–97].

### Type of tracers

The most common tracer used in the studies was technetium-99m radiocolloid (Tc-99mradiocolloid). The second most common tracer was a fluorescent tracer named indocyanine green (ICG). Overall, the calculated detection rate, when using Tc-99m radiocolloid only, was 81% [95% CI: 66–91] compared to 84%[95% CI: 67–93] when using indocyanine green (either with Tc-99m radiocolloid or alone), whereas a combination of Tc-99m radiocolloid and Blue dye resulted in a detection rate of 95%[95% CI: 84–99]. However, when Blue dye was considered as the tracer alone, detection rate appeared to be lower compared to other tracers and the detection rate was calculated as 60% [95% CI: 44–73].

### Site of injection

**FIGURE 4:**
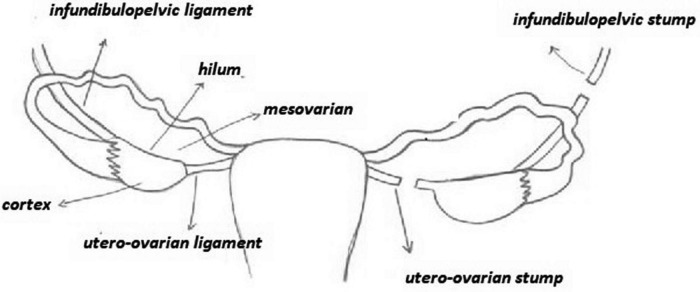
Different sites of tracer injection.

Figure 4 shows different tracer injection sites. The most common injection sites in the patients were proper ovarian and suspensory ligaments. These injections were performed on the dorsal and ventral side of the ovarian ligaments, close to the ovary and just underneath the peritoneum. The second most common site was the mesovarium, followed by the hilum of the ovary, and the ovarian cortex. Hassanzadeh et al. (9) selected two sites for injection of Tc-99mPhytate in patients with ovarian mass. Overall, detection rates for the cases where the injection site was just underneath the peritoneum ovarian ligaments, mesovarium, and ovarian hilum were 85 [95% CI: 71–92], 91 [95% CI: 67–98], and 93% [95% CI: 67–99], respectively. These appeared to have higher detection rates as compared to the cases where the injection was into the ovarian cortex (detection rate: 62% [95% CI: 26–88]).

A number of three studies by Lago et al. (10, 11) and Laven et al. (12) considered an overall total of 41 patients with tracer injection after ovarian mass resection during the same or a subsequent surgical procedure. In other words, the patients underwent laparotomy with frozen section for a pelvic mass suspicious for malignancy or a second staging laparotomy after prior resection of a malignant ovarian mass. The injections were performed at the ipsilateral or bilateral infundibulopelvic and utero-ovarian ligament stumps for unilateral or bilateral tumors with no previous hysterectomy, respectively. An injection was performed only at the infundibulopelvic stump if a hysterectomy had been previously performed. SLN detection rates were 100% in the Lago et al.’s studies (10, 11), but the reported detection rate was much lower (27%) by Laven et al. (12). However, overall, pooled detection rate for the cases (with the injection site in the ligament stumps) was 59% [95% CI: 32–81].

### Tracer injection in normal ovary vs. ovary with underlying pathology

There were two groups of patients in the included studies: a group with normal ovary (e.g., cases of endometrial, cervix cancers, or fallopian tube tumor) and another group which included the patients with underlying ovarian pathology (e.g., ovarian mass or early ovarian cancer). We observed that the detection rates were 91 [95% CI: 75–97] and 82% [95% CI: 70–89] in the normal and abnormal ovary groups, respectively.

### Procedure of surgery (laparotomy vs. laparoscopy)

There were two groups of studies in our systematic review according to the type of surgery (laparotomy vs. laparoscopy). We compared ovarian sentinel lymph node detection rate based on the procedure of surgery, which was calculated as 80[95% CI: 61–91] and 88% [95% CI: 77–95] for laparoscopy and laparotomy, respectively.

### Time between tracer injection and start of sentinel lymph node mapping

The majority of the studies considered 15 min as the median time between tracer injection and sentinel lymph node mapping (visually for Blue dye and by NIR/ICG system and handed gamma probe for ICG and Tc-99m radiocolloid tracer, respectively) (9–19). However, Angelucci et al. (20) considered only 2 min before the start of sentinel lymph node mapping. Also, Buda et al. (21) injected the tracer over the course of laparoscopic surgery and conducted a real-time mapping. However, the last two studies applied ICG as the tracer, which needs less time for lymphatic uptake. In one study (22), there was a reported 4- to 6-h waiting time after injection in lymphoscintigraphy analysis. In the Ataei et al.’s (19) and Speth et al.’s (16) studies, in addition to intraoperative SLN mapping, SPECT/CT lymphoscintigraphy was performed 24 h postsurgery depending on the patients’ conditions.

### Sentinel lymph node location

Table 3 shows location categorization of the ovarian SLNs. Among patients with identified sentinel lymph nodes (*N* = 189), 55% (*N* = 104) were located in the para-aortic region only; 11% (*N* = 21) in the pelvic region only; and 64% (*N* = 34) in both the para-aortic and pelvic region. In the study of Lago et al. (10), SLN locations were not categorized clearly. As such, we did not include this study in the calculation of sentinel node locations. A number of six studies (13–15, 17, 18, 21) reported the location of sentinel lymph nodes to be above or beneath the inferior mesenteric artery (IMA) level and categorized percent location of sentinel lymph nodes related to each of right or left side’s ovary (Table 3). Of 130 patients with unilateral ovary injection in whom the side location of the sentinel lymph nodes was recorded (11–15, 17, 18, 21, 22), in 109 (84%) patients, sentinel nodes were identified only ipsilaterally, in 14 patients (11%), sentinel nodes were identified only contralaterally, and in 7 patients (5%), sentinel nodes were detected bilaterally. In the study of Speth (16), intraoperative gamma-probing SLN mapping was compared with detection by postoperative SPECT/CT imaging. In 4 patients, one or more hotspots could still be identified at locations where the SNs were resected (3 aortocaval and 2 pelvic). Furthermore, in 6 patients, hotspots were detected in pelvic regions that were not identified during surgery. Also, Ataei et al. (19) detected three aberrant locations of sentinel lymph node.

**TABLE 3:** Location of the ovarian Sentinal Lymph Nodes.

First author SLN location	Number of patient with detected SLN	Both pelvic/aortic regions	Pelvic region only	Aortic region only	aortic SLN location to level of the IMA	Location of aortic SLNs of the left and right ovaries
Vanneuville et al. (22)	12	8 (67%)	0	4 (33%)	NA	NA
Negishi et al. (13)	11	4 (36%)	0	7 (64%)	a-IMA = 91% b-IMA = 36% ar-IMA = 36%	Lt ovary: limited to PA region specially a-IMA Rt ovary: at the level of PA to b-IMA
Nyberg et al. (14)	15	0	0	15 (100%)	a-IMA = 33% b-IMA = 67%	Lt ovary: a-IMA 64% Rt ovary: b-IMA 94%
Kleppe (15)	21	5 (24%)	2 (9.5%)	14 (67%)	NA	Lt ovary: a-IMA 100% Rt ovary: ar-IMA 100%
Hassanzadeh et al. (9)	25	2 (8%)	2 (8%)	21 (84%)	NA	NA
Angelucci et al. (20)	5	2 (40%)	1 (20%)	2 (40%)	NA	NA
Buda et al. (21)	9	2 (22%)	1 (11%)	6 (67%)	a-IMA = 22% b-IMA = 42%	Lt ovary: a-IMA 45%, b-IMA 55% Rt ovary: b-IMA/ar-IMA 100%
Speth et al. (16)	3	0	1 (33%)	2 (67%)	NA	NA
Nyberg et al. (17)	20	6 (30%)	2 (10%)	12 (60%)	a-IMA = 33% b-IMA = 67%	Lt ovary: a-IMA 64% Rt ovary: b-IMA 94%
Lago et al. (10)	10	NA	NA	NA	NA	NA
Uccella et al. (18)	21	4 (19%)	4 (19%)	13(62%)	a-IMA = 14% b-IMA = 52% ar-IMA = 14%	NA
Lago et al. (11)	20	19 (95%)	0	1 (5%)	NA	NA
Laven et al. (12)	3	1 (9%)	0	2 (18%)		
Ataei et al. (19)	24	11 (46%)	8 (33%)	5 (21%)	NA	NA
Total	189	64 (34%)	21 (11%)	104 (55%)		

Lt, left; Rt, right; PA, para-aortic; a-IMA, above inferior mesenteric artery; b-IMA, below inferior mesenteric artery; ar-IMA, around inferior mesenteric artery; NA, not available.

### Sensitivity

Only five studies (9, 10, 15, 17, 18) had enough data to calculate the sensitivity of sentinel node mapping in lymphatic staging of ovarian malignancies. The proportion of lymph node-positive patients was 0 to 25% in these studies with overall 14.28%.

**FIGURE 5:**
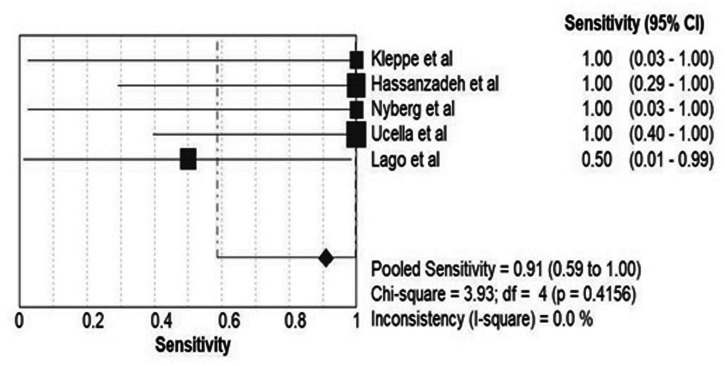
Pooled sensitivity of the included studies for detection of involved nodes.

Figure 5 shows the forest plot of sensitivity pooling across these studies. Overall, pooled sensitivity was 91% [59–100] (Cochrane Q-value = 3.93; *p* = 0.41; I^2^ = 0%).

## Discussion

We conducted a systematic review and meta-analysis of the literature regarding the feasibility of sentinel node mapping and biopsy in ovarian tumors. There have been other reviews on this topic including Uccella et al. (23) and Mach et al. (24). Both studies reported acceptable detection rate and sensitivity for the technique. In addition, both acknowledged that the available evidence is still scarce. We further evaluated the factors that affect the success of sentinel node mapping which we described in the following sections of the discussion.

### Summary of main findings

As mentioned before, the presence of lymph node metastases in early ovarian cancer upstages the disease (7, 10, 18). On the other hand, systemic lymphadenectomy for staging has a high risk of complications (6, 25, 26). Thus, the need for ovarian sentinel lymph node mapping in early ovarian cancer is evidently clear. The present review illustrates that sentinel node mapping of the ovarian masses sounds plausible with an overall detection rate of 86% [95% CI: 75–93] and can vary depending on the type of tracer, site of injection, etc.

As for the type of tracer, detection rate of Blue dye appeared to be lower compared to Tc-99m radiocolloid and indocyanine green. On the other hand, allergic reactions to Blue dye during SLN mapping have been reported in up to 2% of patients (range from urticarial reactions to severe anaphylaxis). In addition, sentinel node mapping using Blue dye seems to be more affected with high BMI as reported by Neyberg et al. (17). In other words, the probability of Blue dye visibility in SLNs with BMI ≥ 27 is lower compared to BMI < 27. One of the advantages of ICG compared to Blue dye is its faster migration to the SLNs as well as better tissue penetration of NIR light compared to normal light. This allows for earlier and deeper detection of the SLNs (27). However, near-infrared fluorescent technology is not available in all centers. Assuming a median net activity of 19 MBq (0.5 mci) per patient, an effective radiation dose equivalent to a chest X-ray (less than 0.1 mSv) is calculated. This implies that the patient is exposed to a small amount of radiation. Alternatively, among three investigated tracers, we observed a maximum SLN detection rate corresponding to the combination of Tc-99m radiocolloid and Blue dye. Therefore, this seems to be the preferred method for ovarian SLN mapping. Interval between tracer injection and lymph node harvesting or mapping can also affect the results of sentinel node mapping. Using Tc-99m radiocolloid, a median 15-min interval seems to have an acceptable SLN detection rate. In contrast, ICG requires a shorter time interval for lymphatic uptake, implying that even a 2-min interval between injections and SLN mapping would yield a high detection rate (20).

It should be mentioned that other radioactive and non-radioactive tracers have also been used for sentinel node mapping in various malignancies. Tc-99m labeled ICG is a very commonly used tracer for sentinel node mapping. In addition to radioactivity guided mapping of the lymph nodes, a visual assistance using near infrared light is used which can help the surgeons in the operative theatre to identify the sentinel nodes more efficiently. However, the need for dimming of the light as well as requiring near infrared cameras can hamper the use of this tracer (28–30). Magnetic materials such as superparamagnetic iron oxide (SPIO) nanoparticles have also been used for sentinel node mapping in different malignancies. The advantage of these tracers is the lack of radiation, and more flexibility regarding the time of injection of the tracer in addition pre-operative imaging is possible (31–34).

The majority of studies have used injection of tracer in the suspensory and the infundibulopelvic ligaments, which showed a pooled detection rate of 85% [95% CI: 71–92]. In the Hassanzadeh et al.’s (9) study, all cases with failed SLN detection were corresponding to the patients with ovarian torsion in addition to their underlying pathology in the ovary. After omitting these four patients, injection of the tracer in the in the suspensory and the infundibulopelvic ligaments showed SLN detection rate of 93%. When observing the correlation between site of injection and detection rate, we recognized that an injection just underneath the peritoneum (ovarian ligaments, mesovarium, and ovarian hilum) resulted in higher detection rate compared to an injection into the ovarian cortex.

Location of sentinel nodes was reported clearly in all but one of the included studies (10). Overall, detection in the aortic region was 55%, whereas the corresponding detection rates for the pelvis and both pelvic/aortic regions were 11 and 34%, respectively. This is in line with the findings of another study which reported that in stage I–II ovarian cancer, lymph node metastases are detected as isolated para-aortic nodes in 50% of patients, in the pelvic region in 20% of patients, and in both the para-aortic and the pelvic region in the remaining 30% (4, 35). This study also concluded that in the majority of cases, aortic region lymphatic drainage of the right ovary is below IMA and that of the left ovary is above IMA. In the patients with unilateral ovary injection in whom the side location of the sentinel lymph nodes was recorded, SLNs were identified in 11 and 5% of cases contralaterally and bilaterally, respectively.

Type of surgery was also an important variable in the included studies (LPS vs. LPT). Laparoscopic performance of sentinel lymph node technique in ovarian cancer seems to be feasible (36). We assessed the SLN detection rate in all studies that were based on the surgical procedure (LPT vs. LPS). SLN detection rate in the LPT procedure group was 88%[95% CI: 77–95] compared to 80% [95% CI: 61–91]in the LPS group.

In most of the studies, tracer injection was performed with the adnexa still *in situ*, with the exception of three studies (10–12). Unlike other studies, these three studies performed injection of the tracer in the infundibulopelvic and utero-ovarian ligament stumps after the removal of the ovarian tumor, during the same or a subsequent surgical procedure. In such cases, the reliability could be lower due to alteration of the lymphatic drainage after ovarian mass resection. Laven et al. (12) reported that “SLN procedure after (previous) resection of the tumor seems inferior to detect sentinel nodes when compared to injection of the tracer in the ovarian ligaments before tumor resection.” However, SLN detection rates were 100% in the Lago et al.’s studies (10, 11). As such, overall, detection rate for these cases (with the injection site in the ligament stumps) was 59% [95% CI: 32–81]. Overall, 41 patients were included in the three aforementioned studies, and further studies are needed to explore the impact of tracer injection after ovarian mass resection in the ovarian SLN mapping.

A few studies (9, 10, 15, 17, 18) had enough data to calculate the sensitivity of sentinel node mapping in lymphatic staging of ovarian malignancies. Overall, pooled sensitivity was 91% [59–100], which seems to be an acceptable sensitivity for ovarian SLN mapping.

### Limitations

Definitive diagnosis of benign or malignant ovarian mass prior to resection is not determined, which is a major limitation in the ovarian SLN mapping. Therefore, patients with malignant ovarian mass who were included in these studies were not enough for lymph node dissection and histopathological examination regarding false-negative rate and sensitivity assessment. As such, further studies are needed to more accurately determine the sensitivity of sentinel node mapping in lymphatic staging of ovarian malignancies.

## Conclusion

Sentinel node mapping in ovarian tumors is feasible and seems to have high sensitivity for the detection of lymph node involvement in malignant tumors of ovary. Mapping material, injection site, and previous surgery of the ovaries were associated with successful mapping. Larger studies are still needed to better evaluate the sensitivity of this procedure in ovarian malignancies.

## Data availability statement

The original contributions presented in this study are included in the article/supplementary material, further inquiries can be directed to the corresponding author.

## Author contributions

SA and RS: study design. SA, MF, MH, and RS: data acquisition. SA, SM, and RS: quality control of data and algorithms and manuscript preparation. RS and SA: data analysis and interpretation. RS: statistical analysis. All authors: study the concepts, editing and review the manuscript. All authors have read and agreed to the published version of the manuscript.

## Footnotes

1
http://www.cebm.net/index.aspx?o=5653

2
http://www.prisma-statement.org/PRISMAStatement/
